# Incidental finding of tuberculous pleural effusion in patient undergoing coronary artery bypass grafting: Case report

**DOI:** 10.1016/j.amsu.2019.08.001

**Published:** 2019-08-09

**Authors:** Yasir Khan, Munawar Shah, Syed Shahabuddin

**Affiliations:** Section of Cardiothoracic, Department of Surgery, Aga Khan University Hospital Karachi, Pakistan

**Keywords:** Tuberculous pleural effusion, Coronary artery bypass grafting

## Abstract

**Introduction:**

Pleural effusion is common in patient with coronary artery bypass grafting (CABG) usually due to heart failure in preoperative and trauma of surgery in postoperative patient. Tuberculous Pleural effusion is most common form of extra pulmonary Tuberculosis. Preoperatively Tuberculous pleural effusion in CABG patients has rarely been described in literature.

**Presentation of case:**

A 62 years old gentleman with ischemic heart disease was admitted for coronary artery bypass grafting surgery. Preoperative workup showed left sided pleural. Intraoperatively left sided turbid yellowish colour effusion with loculation was noted while harvesting left internal mammary artery. Loculation were broken down, effusion was drained and tissues were sent for microbiology and histopathology. CABG was performed smoothly. Microbiology of pleural tissue revealed *Mycobacterium tuberculosis* while histopathology showed chronic granulomatous inflammation. Patient was started on antituberculous therapy and remained well six months postoperatively.

**Conclusion:**

In developing countries even without any constitutional symptoms of Tuberculosis high index of suspicion for Tuberculosis should be made for patient with pleural effusion especially in cases of cardiac surgery as to prevent morbidity and mortality.

## Highlights

• Tuberculosis is a Global health problem.
• Pleural effusion in developing country should be evaluated for Tuberculosis.
• Pleural effusion in patients with Coronary artery disease is not always due to heart failure, it can be due to Tuberculosis [1-8].

## 1 Introduction

The following case report has been reported from Our University Hospital which is an internationally recognized teaching hospital and a tertiary care center based in Pakistan, in accordance with the SCARE guidelines for case reports [1]. Tuberculosis, one of the significant world health burdens of both developed and developing countries, with around 9.6 million cases a year [2]. Tuberculous pleural effusion (TPE) is the most common form of extra pulmonary tuberculosis [3]. TPE results from *Mycobacterium tuberculosis* infection of the pleura and is characterized by an intense chronic accumulation of fluid and inflammatory cells in pleural space^2;^ resulting in significant morbidity and mortality. Pleural effusion is common in patient with coronary artery bypass grafting (CABG) usually due to heart failure in preoperative [4] and trauma of surgery in postoperative patient [5]. Tuberculous pleural effusion and pericardial effusion has been described in literature post CABG but tuberculous pleural effusion in preoperative patient is rarely described [6]. In majority of CABG patients preoperative pleural effusion is not analyzed as its mostly consider to be due to heart failure but timely recognition of such patient with infection especially chronic infection can prevent significant morbidity and mortality. We report a case of 62 years old gentleman who came to us for CABG with left sided pleural effusion that turned out to be Tuberculosis.

## 2 Presentation of case

**Fig. 1:**
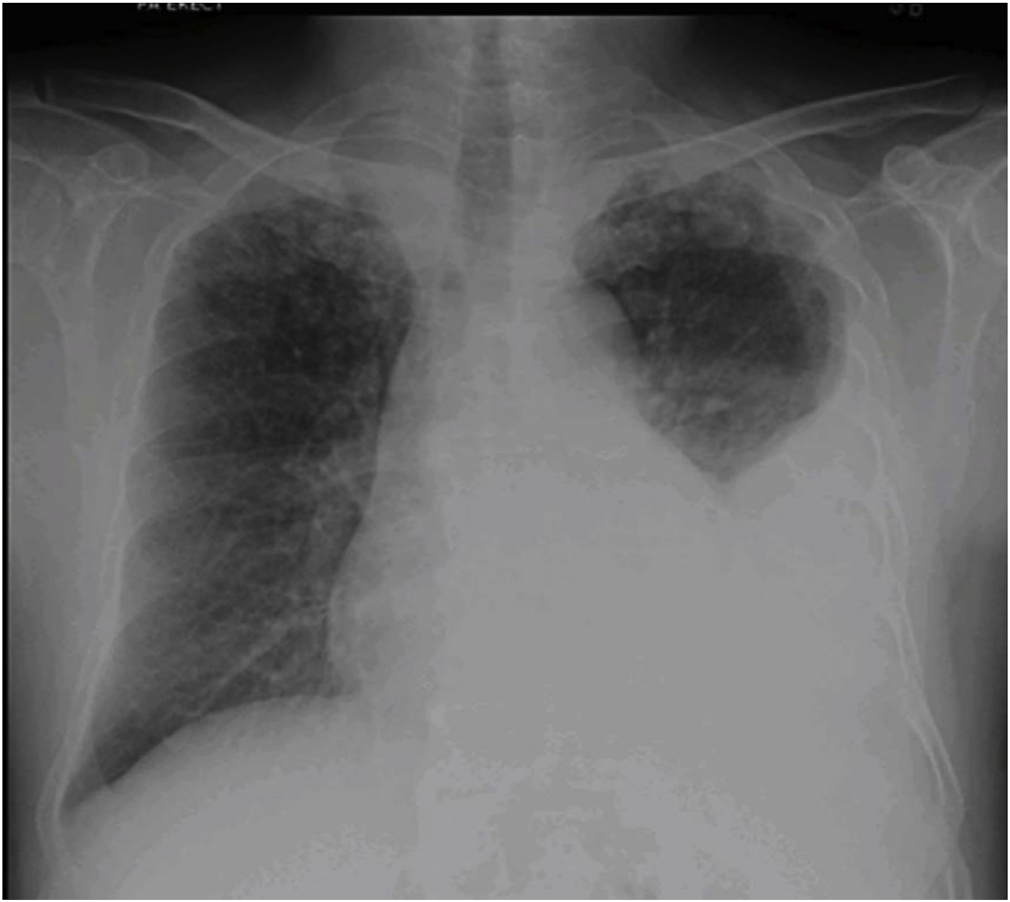
Chest x ray preoperatively showing left sided effusion.

A 62 years old gentleman presented with recent history of non ST elevated myocardial infarction (NSTEMI) having persistent chest discomfort and dyspnea on exertion. His blood workup, radiological examination was unremarkable. Angiography showed critical coronary artery disease with normal functioning left ventricle on echocardiography (ECHO). He was advised urgent CABG considering his angiography. Due to financial constrain patient presented about 2 and half week latter for CABG. Pre-CABG workup was performed. His biochemistry and blood workup was unremarkable except for mild raise in monocytes. On Chest X-ray left sided effusion was noted (Fig. 1). There was no history of fever and other constitutional symptoms. CABG x 4 was performed with left internal mammary artery to left anterior descending artery by senior surgeon. Intraoperative left sided loculated effusion was noticed with turbid fluid. Fluid was drained, Loculation broke down and pleural tissue was sent for microbiology and histopathology. Postoperatively patient remained stable and was discharged. His final culture was positive for Acid Fast Bacillus and histopathology revealed granulomatous disease. Patient was referred to infectious disease service for antituberculous therapy and six month follow-up patient remained well.

## 3 Discussion

Pleural effusion in heart disease patient is relatively common phenomenon. Preoperatively common cause of pleural effusion in coronary artery disease is acute cardiogenic pulmonary secondary to sever myocardial dysfunction/infarction [7]. Recognition of such patients is important as it carries high risk of mortality. While postoperatively trauma of surgery or postcardiotomy syndrome is common cause of pleural effusion [4,5]. Post cardiotomy syndrome is associated with fever and chest pain. Simple pleural effusion post coronary artery bypass grafting is common and is divided into early or late. Early pleural effusion is usually due to trauma of surgery and is hemorrhagic. Treatment of these effusions is conservative or thoracocentesis [5].

TPE usually manifest as acute illness with majority of patient complaining of pleuritic chest pain, fever and cough. Rarely it is asymptomatic [2,3]. Upon presentation symptoms may be present for less than a month or less than a week. TPE is mostly unilateral 98% and more on right side 56%. 20% of cases there is associated lung parenchymal disease. Definite diagnosis is made by mycobacterium in sputum, pleural fluid and pleural biopsy [3].

TPE in CABG patients is rarely been describe and in most of these case TPE developed postoperative [6]. Patients undergoing CABG have myocardial ischemia and as such CABG carries a significant risk of morbidity. Presence of simultaneous preoperative tuberculosis and myocardial ischemia in patients undergoing CABG can lead to significant morbidity and mortality. Therefore timely recognition of any infection especially chronic infection like tuberculosis is of paramount importance. Tuberculous pleural effusion is a global problem but its incidence has been decrease in developed countries however TPE is still high in developing countries with majority present in south Asian population [8].

Here we describe a case of acute pleural effusion in patient with critical coronary artery disease requiring coronary artery bypass grafting without any constitutional symptoms turning out to be Tuberculosis is a significant entity. Importance of the case is timely recognition of Tuberculosis in such high risk patient. In south Asian population where tuberculous pleural effusion is still common, high index of TPE suspicion should be kept in preoperative pleural effusion. With timely recognition of TPE major morbidity and mortality can be avoided especially in CABG patients.

## 4 Conclusion

Hence we recommend pleural biopsy/AFB microbiology for patient with preoperative pleural effusion requiring CABG without signs of heart failure especially in developing countries.

## Disclosure

No funding is associated with this research.

## Consent

Informed consent was obtained from the patient for publication of this case report and accompanying images.

## Provenance and peer review

Not commissioned, externally peer reviewed.

## Ethical approval

Departmental consent taken.

## Sources of funding

None.

## Author contribution

• Yasir Khan – Study concept, study design, review of literature, manuscript writing.
• Munawar Hussain – Data interpretation, proof reading.
• Syed Shahabuddin– Data analysis, interpretation, manuscript drafting

## Registration of research studies

1. Name of the registry: not required
2. Unique Identifying number or registration ID:
3. Hyperlink to the registration (must be publicly accessible):

## Guarantor

Syed Shahabuddin.

Assistant Professor Cardiothoracic Surgery.

Aga Khan University Hospital Karachi, Pakistan.
